# Use of telephone and SMS reminders to improve attendance at hospital appointments: a systematic review

**DOI:** 10.1258/jtt.2011.110707

**Published:** 2011-10

**Authors:** Per E Hasvold, Richard Wootton

**Affiliations:** Norwegian Centre for Integrated Care and Telemedicine, University Hospital of North Norway, Tromsø, Norway

## Abstract

Patients failing to attend hospital appointments contribute to inefficient use of resources. We conducted a systematic review of studies providing a reminder to patients by phone, short message service (SMS) or automated phone calls. A PubMed search was conducted to identify articles published after 1999, describing studies of non-attendance at hospital appointments. In addition, we searched the references in the included papers. In total, 29 studies were included in the review. Four had two intervention arms which were treated as independent studies, giving a total of 33 estimates. The papers were analysed by two observers independently. A study quality score was developed and used to weight the data. Weighted means of the absolute and the relative changes in non-attendance were calculated. All studies except one reported a benefit from sending reminders to patients prior to their appointment. The synthesis suggests that the weighted mean relative change in non-attendance was 34% of the baseline non-attendance rate. Automated reminders were less effective than manual phone calls (29% vs 39% of baseline value). There appeared to be no difference in non-attendance rate, whether the reminder was sent the day before the appointment or the week before. Cost and savings were not measured formally in any of the papers, but almost half of them included cost estimates. The average cost of using either SMS, automated phone calls or phone calls was €0.41 per reminder. Although formal evidence of cost-effectiveness is lacking, the implication of the review is that all hospitals should consider using automated reminders to reduce non-attendance at appointments.

## Introduction

Non-attendance for appointments in health care results in wasted resources and disturbs the planned work-schedules. Cancellations and rescheduling of appointments are usually dealt with administratively and vacant slots are often filled by other patients, which reduces the loss in overall efficiency for the health-care staff concerned. In hospitals, the problem of non-attendance can be met by a number of different strategies, such as overbooking the appointment list or sending some kind of reminder in advance of the appointment. However, overbooking may not be considered an appropriate method in modern health-care delivery. On the other hand, reminders directly to the patient from a hospital are generally acceptable. This can be viewed as a form of telemedicine, since it is an application of technology to the health-care process which involves distance.

It seems reasonable to expect that sending reminders would decrease the no-show rate at hospital appointments. However, there is little information about the magnitude of this effect and we are only aware of one previous review of the effect of reminders on non-attendance at hospital appointments.^1^ This was a narrative review of telephone and postal reminders, which concluded that reminders can improve attendance and reduce non-attendance qualitatively. We have therefore conducted a systematic review. The research questions were:

1. What is the best estimate of the effect of sending reminders on non-attendance rates?
2. Are there any differences in non-attendance when using reminders sent manually (i.e. from phones operated by a human) or automatically (i.e. by SMS text messages or by automated voice recordings)?
3. Does the time at which the reminder is sent influence the effect on non-attendance rates?
4. What are the costs and benefits of using reminders?

## Methods

Papers were selected following the PRISMA methodology.^2^ A search of the PubMed database was conducted on 21 February 2011 using the following keywords:

(telephone OR phone OR mobile OR cellphone) AND (outpatient OR out-patient) AND (attendance* OR appointment OR reminder)

Only papers published in 2000 or later, in English or any of the Scandinavian languages (Danish, Swedish or Norwegian) were included. In addition we examined the reference lists of the papers selected for review. Duplicates were then eliminated. These were screened for relevance, i.e. to confirm that they reported reminders using phones or SMS, leaving papers for full-text eligibility assessment. Papers were eliminated from the study if they provided insufficient data about change in attendance or did not describe reminders for a particular appointment, but general adherence to long-term programmes.

### Data analysis

All papers were analysed by both authors independently. Any disagreements in interpretation were resolved by consensus. Four of the selected papers described multi-arm studies in which reminders were sent both by phone and by SMS, or by phone and by automated phone calls to separate groups. In these cases we used data from both arms of the study as though they were independent studies.

The outcome variable of interest was the Did Not Attend (DNA) rate. When a paper reported that a reminder was sent ‘within a week before the appointment’ the reminder time was assumed to be 3.5 days.

### Study quality

A compound quality indicator was created for weighting the results according to the following indices:

1. Study size (0 = not stated; 1 = 1–100; 2 = 101–1000; 3 = 1001–10000; 4 = >10000);
2. Duration of intervention (0 = duration not stated; 1 = 1–3 months; 2 = 4–12 months; 3 = >12 months);
3. Study design (0 = not stated; 1 = retrospective controls; 2 = before and after, or non-randomized control study; 3 = RCT). Note that in a retrospective trial, the baseline may have been measured in the year before the intervention, i.e. there would have been an interval before the intervention started. In a before and after study, the intervention starts immediately after the baseline has been measured;
4. Cost of intervention (0 = not stated; 1 = estimate of costs; 2 = measurements of costs according to current guidelines for economic evaluation in health care^32^);
5. Savings from intervention (0 = not stated; 1 = estimate of savings; 2 = measurements of savings according to current guidelines^32^).

This gave a possible score for study quality from zero to 14. Similar quality indicators have been used previously by others.^33,34^

### Effect size

Two effects were examined: the absolute and the relative change in DNA rate. The absolute change in the DNA rate was calculated as the percentage of DNA in the control group minus the percentage of DNA in the intervention group. The relative change in the DNA rate was calculated by dividing the absolute change by the percentage of DNA in the control group.

### Pooled estimate of effect size

Weighted mean values were calculated using the quality scores as weights.

### Data extraction

In papers reporting the DNA rates only, we did not attempt to contact the authors for clarification or additional information about their data. We checked the numbers of patients involved and recalculated the rates to four significant figures based on an integer number of patients. We analysed the data on an ‘intention to treat’ basis.

We categorised the interventions as manual or automated. Manual reminders were telephone calls made by members of staff. Automated calls were either computer driven voice messages or computer driven SMS text messages.

We categorised the ages of the patients as child (neonatal/paediatric/adolescent) (0–17 years), adult (18–60 years) or geriatric (>60 years). In some cases the age of the patients was unclear and we assumed it to be adult.

## Results

**Figure 1:**
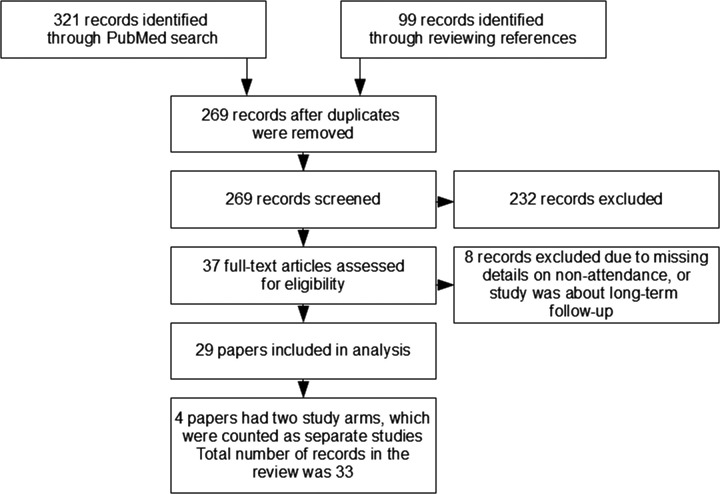
The PRISMA flowchart for the paper selection process

The search returned 321 records. The reference lists of relevant papers (see below) produced another 99 records. After duplicates were eliminated there were 269 records. These were screened for relevance and the screening eliminated 232 papers, leaving 37 papers for full-text eligibility assessment. Of these, eight papers were eliminated from the study, which left 29 papers for full analysis, see Table 1.^3–31^ Figure 1 shows the PRISMA flowchart of the selection process.

**Table 1:** Papers selected for review

Study	Reminder type (manual or automatic)	Study size	Country	Study design	Baseline DNA %	Intervention DNA%
Adams, 2004	Manual	2823	Australia	Telephone reminders for 3 months; retrospective comparison with previous year	12.2	9.0
Booth, 2004	Manual	100	UK	Telephone reminders for 4 months; concurrent and matched groups	40.0	14.0
Bos, 2005	Manual Automatic	216	Netherlands	Telephone and SMS reminders for 0.75 months; concurrent groups	6.5	M: 2.7 A: 2.0
Chen, 2008	Manual Automatic	1848	China	Telephone and SMS reminders for 2 months; RCT	19.6	M: 11.7 A: 12.5
Corfield, 2008	Manual	1077	UK	Telephone reminders for 2 months; retrospective control group	21.4	19.7
da Costa, 2010	Automatic	29014	Brazil	SMS reminders for 11 months; concurrent, non-randomized (patients who accepted SMS were sorted into the intervention group)	25.6	19.4
Dockery, 2001	Manual	162	UK	Telephone reminders for 2 months; before and after study	29.5	17.9
Downer, 2005	Automatic	2864	Australia	SMS reminders for 1 month; retrospective comparison with previous month	23.4	14.2
Downer, 2006	Automatic	45110	Australia	SMS reminders for 3 months; retrospective comparison with previous year	19.5	9.8
Foley, 2009	Automatic	709	UK	SMS reminders for 1 month; retrospective comparison with previous year	23.9	10.4
Geraghty, 2007	Automatic	8966	Ireland	SMS reminders for 36 months; historical control group consisted of patients not sent SMS in the intervention period	33.6	22.0
Hardy, 2001	Manual	325	UK	Telephone reminders; duration not stated; single centre, prospective, non-randomized, controlled study	7.3	1.4
Hashim, 2001	Manual	823	USA	Telephone reminders for 1 month; RCT	25.6	19.8
Haynes, 2006	Manual	515	USA	Telephone reminders for 7 months; non-randomized controlled study	11.6	4.7
Irigoyen, 2000	Manual	653	USA	Telephone reminders for 5 months; non-randomized controlled trial	35.0	34.9
Koshy, 2008	Automatic	9959	UK	SMS reminders for 6 months; non-randomized controlled trial	18.1	11.2
Kruse, 2009	Automatic	1027	Denmark	SMS reminders for 1 month; prospective cohort study	10.0	5.9
Lee, 2003	Manual	161	Ireland	Telephone reminders for 2 months; before and after study	23.3	5.7
Leong, 2006	Manual Automatic	993	Malaysia	Telephone and SMS reminders for 7 months; RCT	51.9	M: 40.4 A: 41.0
MacDonald, 2000	Manual	719	New Zealand	Telephone reminders for 36 months; non-randomized controlled study	24.4	18.4
Maxwell, 2001	Automatic	1370	USA	SMS reminders for 2 months; RCT	40.0	36.9
McPhail 2010	Automatic	145	USA	SMS reminders for 12 months; non-randomised controlled study?	72.5	20.4
Milne, 2006	Automatic	16400	UK	SMS reminders for 2 months; retrospective study	15.4	12.0
Parikh, 2010	Manual Automatic	9835	USA	Telephone and SMS reminders for 5 months; RCT	23.1	M: 13.6 A: 17.3
Perron, 2010	Manual	2123	Switzerland	Telephone reminders for 3 months; RCT	11.4	7.8
Reti, 2003	Manual	74	New Zealand	Telephone reminders for 3 months; RCT	27.0	8.1
Roberts, 2007	Manual	504	UK	Telephone reminders for 10 months; RCT	20.9	13.8
Satiani, 2009	Automatic	8766	USA	SMS reminders for 17 months; non-randomized controlled study	5.9	8.9
Sawyer, 2002	Manual	171	Australia	Telephone reminders for 6 months; RCT	20.0	7.9

### Analysis

The analysis below is based on data from 29 studies reporting a total of 33 estimates. Eighteen of the interventions were based on manual reminders (i.e. phone calls made by health staff) and 15 were based on automated reminders (i.e. automated phone messages or SMS messages). The study characteristics are summarised in Table 2.

**Table 2:** Study characteristics

	Median	Lower quartile	Upper quartile
Study size	823	325	2864
Duration of intervention (months)	3	2	7
Reminder time (days before appointment)	2.75	1.00	3.13

The median DNA rate reported at baseline (i.e. in the control group) was 23%. All studies except one reported that the intervention improved the DNA rate. The median DNA rate reported after the intervention was 13%, see Table 3.

**Table 3:** DNA rates reported in 29 studies (33 estimates), unweighted

	Median	Lower quartile	Upper quartile
Baseline DNA rate (%)	23.1	15.4	27.0
Intervention DNA rate (%)	12.5	8.1	19.4
Absolute change in DNA rate (%)	7.0	4.2	11.5
Relative change (% of baseline value)	38.1	24.1	58.0

**Figure 2:**
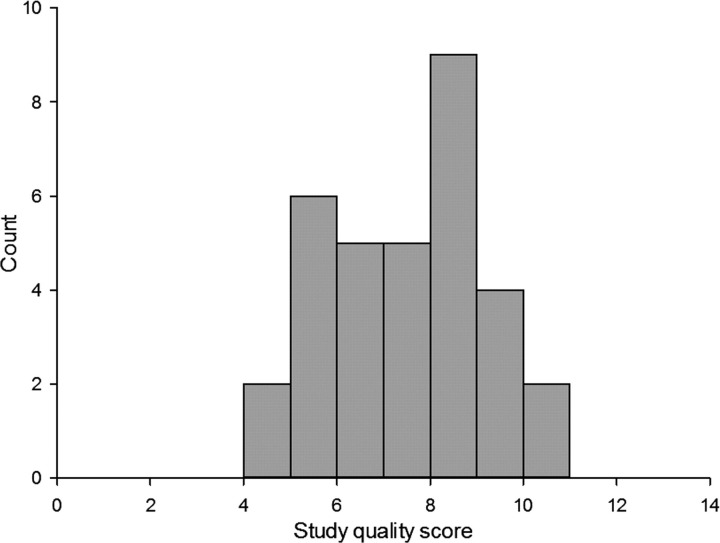
Study quality (median quality score = 7)

Nine of the 29 studies were randomised controlled trials. The median study quality score was seven, see Figure 2.

**Figure 3:**
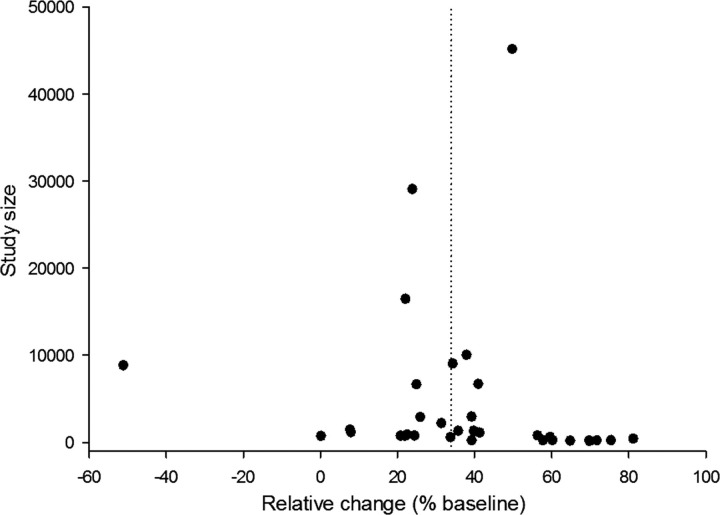
Funnel plot of relative change in DNA rate (% of baseline value)

There was little evidence for publication bias based on a funnel plot, see Figure 3.

**Figure 4:**
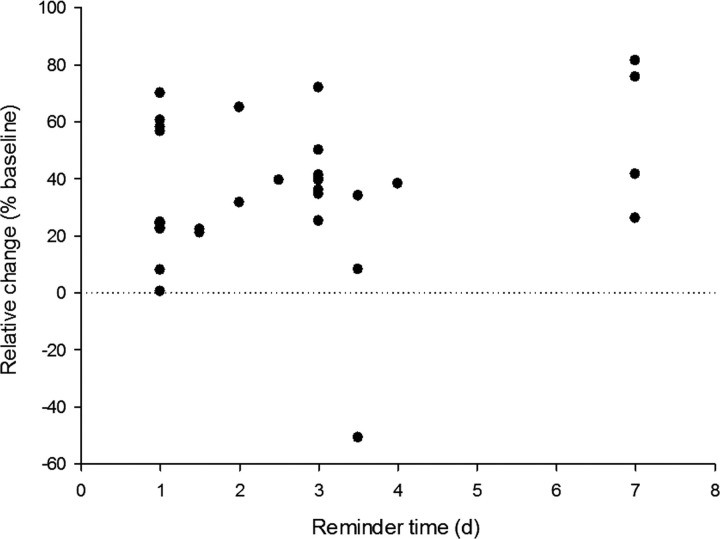
Effect size (relative change in DNA rate) and the time at which the reminder was issued

**Figure 5:**
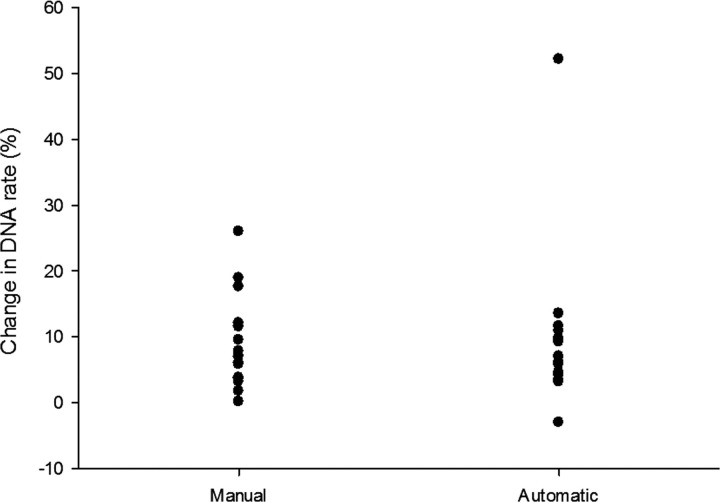
Absolute change in DNA for manual and automated (SMS or automated phone call) reminders

**Figure 6:**
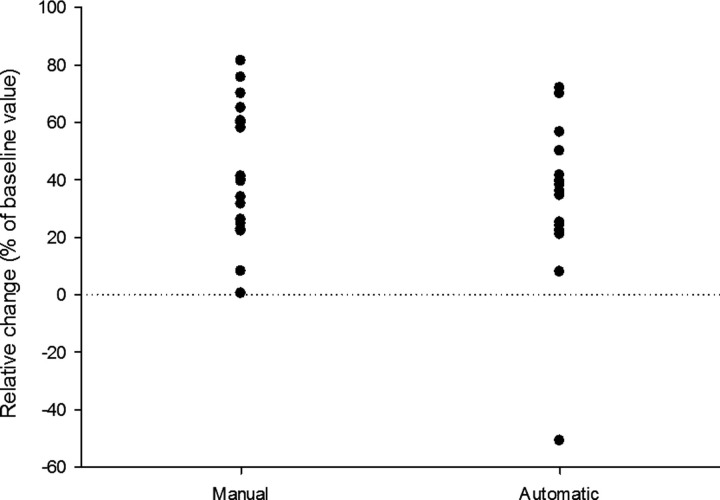
Relative change in DNA (% of baseline) for manual and automated (SMS or automated phone call) reminders

There was no obvious relation between effect size and the time at which the reminder was issued, see Figure 4 (Spearman correlation 0.18). Three of the 29 studies stated that the reminder was sent within a week of the appointment, which we assumed to be within 3.5 days for the purposes of analysis. The effect of reminders on DNA rates was higher for manual reminder calls than for automated calls; this was true for both the absolute and the relative change in DNA rates, see Figure 5 and 6 respectively.

The mean estimates of effect size are summarised in Table 4.

**Table 4:** Pooled estimates

	No of estimates	Weighted mean	Unweighted mean
Manual reminders			
absolute change in DNA rate (%)	18	8.3	8.9
relative change in DNA rate (% baseline)	18	39.1	42.2
Automated reminders			
absolute change in DNA rate (%)	15	8.9	9.7
relative change in DNA rate (% baseline)	15	28.9	32.5

### Costs and savings

None of the papers included costs and savings data to the standard of accepted guidelines for economic evaluation in health care. An estimate of the cost of the intervention was reported in 16 of the papers. Two of the estimates were not included in the present study as the cost estimates depended on circumstances or cost sharing models that were particular to the case. The cost estimates were converted into Euros, using the exchange rate giving the highest costs for the year the paper was published. The average estimated costs in these 14 studies was € 0.41 per patient. The mean cost of phone reminders was € 0.90, while the mean cost of SMS or automated phone call reminders was € 0.14. The three highest reported costs were from phone reminders.

While savings were estimated in 10 papers, the circumstances and cases could not be compared.

## Discussion

The present study concerns a systematic review of the use of telephone reminders (manual and automated) to improve attendance at hospital appointments. All studies except one found that sending reminders improved DNA rates. This suggests the possibility of publication bias, although we found no evidence that this was the case. Taking into account the quality of the studies, the pooled estimates show that manual reminders can achieve a reduction in the DNA rate of 39% of the baseline value, while automated reminders can achieve a reduction of 29% of the baseline value. It seems intuitive that reminders from a health-care professional would be more effective than those sent automatically by a computer.

Our pooled estimate of effect size was based on a weighted mean, using study quality as the weighting factor. We did not attempt a formal meta-analysis for several reasons. First, only nine of the 29 studies were RCTs. Second, the studies were very heterogeneous. Finally, it was not possible to estimate the SE of the treatment effect from many of the published reports. Most of the papers reported the absolute change in DNA. We used the relative change in DNA to compensate for the different baseline DNA rates in the different settings of the studies.

Although the quality of the economic data was weak, the apparent cost of sending reminders was much lower than the expected savings from avoided missed appointments.

The time between the reminder and the appointment did not seem to have any strong effect on the DNA rate for any of the methods (see Figure 4). All studies involved reminders being sent out within a week, which appears to be an appropriate time ahead of an appointment to avoid people forgetting about it.

An analysis of the relation between the age of the patients and the observed effect showed no difference between the age groups.

### Limitations

In the papers which studied two kinds of reminder simultaneously, we treated the two arms as independent studies since they were separate groups, when there might have been interdependencies.

Our initial search was conducted using a single database and clearly if more databases had been used, more references might have been found. However a study by Bahaadinbeigy *et al.* ^35^ suggests that more than 80% of telemedicine papers can be found by searching in Medline alone. We also conducted a search in the Psycinfo database, using the same terms, but found no additional relevant studies.

All studies except one showed a positive effect from using reminders. The exception was the study by Satiani *et al.* ^30^ This study also differed from the others because the patients themselves chose in advance whether they wished to receive a reminder or not. This may have introduced a bias in the intervention group.

### Further studies

We recommend that rigorous health economics studies of the costs and savings of reminders should be carried out, preferably in the form of randomized controlled trials. Without such studies, it is not possible to know with certainty whether automated reminders such as SMS text messages are better than human-generated telephone calls (which are more expensive, but produce bigger improvements in DNA rates). Future research should also be carried out to investigate the benefits of sending multiple reminders, and whether that leads to ‘reminder fatigue’ in the recipients. Some of the papers in the present review reported that the patients were not necessarily happy about the reminder they had received, despite stating that they would still like to be reminded about any future appointments.

Most papers only provided numbers for attendance or non-attendance. Cancellations and rescheduling were not reported in enough papers to be considered in the analysis, but this is an important aspect of how reminders may contribute to a better and more efficient use of the resources and it is possible that the time of the reminder may have a more interesting effect on cancellations than on non-attendance.

### Conclusions

Sending appointment reminders from hospitals to patients can be seen as a form of telemedicine since it involves distance and is an application of technology which contributes to the health-care process. The evidence is overwhelming that reminders have a positive effect on non-attendance rates. Our study shows that a 39% improvement in the baseline DNA rate can be expected when manual reminders are employed, and a 29% improvement when automated reminders are used. While the costs were only estimated, the studies reviewed suggest that reminders cost less than € 0.50 per patient for SMS or automated reminders. This seems likely to be much less than the cost of missed appointments and we therefore recommend that reminders are used routinely for all hospital appointments.
